# Development and validation of a deep learning model based on cascade mask regional convolutional neural network to noninvasively and accurately identify human round spermatids

**DOI:** 10.1016/j.jare.2025.03.059

**Published:** 2025-04-02

**Authors:** Yujiao Sun, Shihao Shao, Jiangwei Huang, Hao Shi, Liying Yan, Yongjie Lu, Ping Liu, Yuqiang Jiang, Jie Qiao, Li Zhang

**Affiliations:** a Institute of Genetics and Development Biology, Chinese Academy of Sciences, Beijing 100101, China; b School of Basic Medical Sciences Peking University, Beijing 100101, China; c National Center for Protein Sciences at Peking University, School of Life Sciences, Peking University, Beijing 100191, China; d Center for Reproductive Medicine, Department of Obstetrics and Gynecology, Peking University Third Hospital, Beijing 100191, China; e National Clinical Research Center for Obstetrics and Gynecology, Beijing 100191, China; f Key Laboratory of Assisted Reproduction (Peking University), Ministry of Education, Beijing 100191, China; g Beijing Key Laboratory of Reproductive Endocrinology and Assisted Reproductive Technology, Beijing 100191, China; h National Clinical Key Specialty Construction Program, Beijing 100191, China; i Beijing Advanced Innovation Center for Genomics, Beijing 100191, China; j Peking-Tsinghua Center for Life Sciences, Peking University, Beijing 100191, China

**Keywords:** Human round spermatids, Human round spermatid injection, Deep learning, Artificial intelligence, Mask region-based convolutional neural network

## Abstract

**Introduction:**

The difficulty of identifying human round spermatids (hRSs) has impeded applications of the human round spermatid injection (ROSI) technique. RSs can be accurately screened through flow cytometric analysis utilizing the Hoechst fluorescence profile reflecting DNA, but this method is not suitable for isolating hRSs due to the toxicity associated with Hoechst staining.

**Objective:**

To evaluate the capacity of a deep learning model grounded in a cascade mask region-based convolutional neural network (R-CNN) for the noninvasive and accurate identification of hRSs.

**Methods:**

In this study, we presented the development and validation of a deep learning model for identifying hRSs through the analysis of 3457 optical light microscope images of sorted hRSs obtained via flow cytometric analysis. The model’s accuracy and specificity were evaluated by calculating the mean average precision (mAP). Furthermore, a double-blind experiment was conducted to access the reliability of the proposed model in accurately identifying hRSs. It detected the expression of protamine (PRM1) and/or peanut lectin (PNA), which are established markers for RSs.

**Results:**

Our deep learning-based model demonstrated a high precision, achieving a mAP of over 0.80 for isolating hRSs in test datasets. The expression of PRM1 and/or PNA was observed in all cells noninvasively selected by our AI model during an independent double-blind test. This phenomenon confirmed the accuracy and effectiveness of the proposed model. The model’s capability for noninvasive and accurate isolation of hRSs among spermatogenic cells highlighted its robustness and generalizability for clinical applications.

**Conclusion:**

The deep learning AI model based on a cascade R-CNN has the ability to accurately identify hRSs among spermatogenic cells. The application of this noninvasive method, which requires no additional procedures in clinical practice, is able to facilitate the widespread implementation of ROSI technique. Therefore, it can provide patients with spermatogenic arrest the opportunity to become biological fathers.

## Highlights

• Deep learning model was built by analyzing images of sorted human round spermatids (hRSs) by flow cytometric analysis.
• Expression of PRM1and/or PNA (RSs markers) was observed in all cells selected by our model.
• Results of double-blind test proved accuracy and effectiveness of our model for identifying hRSs.
• Our model solved the most difficult technological problem of noninvasively and accurately identifying hRSs.
• Our model will promote widely clinical application of human round spermatid injection technique.

## Graphical abstract

**Figure:**
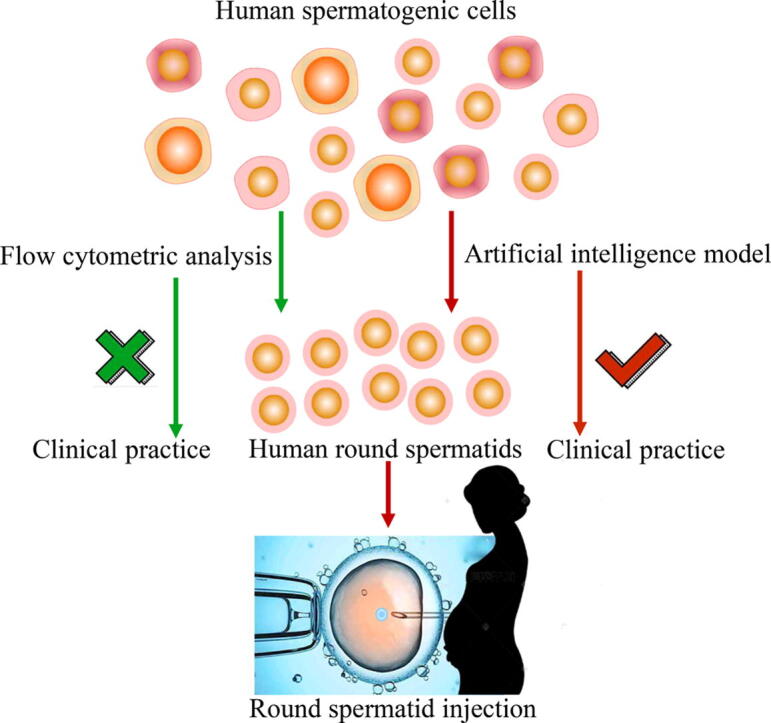

## Introduction

Azoospermia, defined as the absence of sperm in two consecutive semen analyses, is observed in 10–15 % of infertile males. Meanwhile, approximately 60 % of azoospermia cases are diagnosed as non-obstructive azoospermia (NOA), which is primarily attributed to impaired spermatogenesis [1]. Microdessection testicular sperm extraction (mTESE) serves as the primary treatment approach for sperm retrieval in NOA patients, boasting high sperm retrieval rates while minimizing tissue loss [2]. For these patients, intracytoplasmic sperm injection (ICSI) utilizing testicular sperm offers the possibility of biological fathers [3]. The average sperm retrieval rate in NOA males is around 50 %, and about 30 % of them with spermatogenic arrest who don’t have mature sperm but do have haploid hRSs with the same chromosomes number and DNA contents as matured sperm have been observed during mTESE surgeries [4].

Round spermatid injection (ROSI), a form of assisted reproductive technology (ART), have provided spermatogenic arrest patients whom have hRSs as the most mature germ cells the opportunity to obtain offspring [5], [6], [7], [8], [9]. However, widespread implementation of ROSI has been limited since the first human babies conceived through this technique were reported in 1995 [5]. This limitation arises from the imprecise screening methods for hRSs, which primarily rely on the subjective experience of embryologists based on morphological and physical characteristics. Such methods have proven difficult to replicate by other embryologists and are associated with low reproducibility.

To enhance the practicality and robustness of hRSs selection, we proposed an automatic hRSs selection pipeline powered by artificial intelligence (AI) techniques. Deep learning models have demonstrated extraordinary performance in the field of computer vision, such as object detection [10], [11], semantic segmentation [12], [13], [14], and so on. In particular, AI-based models have also shown remarkable success in the medical imaging domain, such as breast tumor detection in ultrasound images [10].We adopt the cascade mask region-based convolutional neural network (Cascade Mask R-CNN) [15] as our base model. The cascade region of interest (RoI) heads strengthens the model’s capacity to accurately model data distribution. Several clinical applications have demonstrated its high performance [16], [17]. However, there remains room to for improvement in its performance [15], [18]. Notably, the application of Cascade Mask R-CNN for hRSs selection is currently limited. Screening hRSs presents a typical dense prediction challenge characterized by an extreme imbalance between positive (hRSs) and negative (background) samples. Diao et al. recognized this issue and proposed a novel way to duplicate the positive region in the images [19]. Inspired by this work, we proposed to elevate the magnitude of both false positive (FP) and false negative (FN) objects by establishing FP Zoo and FN Zoo, termed “Confusion Duplicate”. Additionally, we introduced a Regional Drop operation to efficiently implement “DropBlock” (A popular regularization method for convolutional networks) in both FP and FN regions. This minimized the likelihood of applying DropBlock to non-informative areas. By incorporating the two proposed augmentation strategies above, we have developed a high-performance automated hRSs selection pipeline which was presented in this paper.

In our study, sufficient hRSs were selected using flow cytometry analysis at first. Then, equipped with our novel pipeline, the deep learning model was effectively trained to accurately screen hRSs. The model achieved a mean Average Precision (mAP) of over 0.80 in the test datasets, demonstrating a satisfactory trade-off between precision and recall. Additionally, our proposed augmentation strategies consistently outperformed the baseline model by approximately 0.054 in terms of mAP. Furthermore, the expression of round spermatid markers, including protamine (PRM1) and/or peanut lectin (PNA) [20] was observed in all cells selected by our AI model, confirming its high practicality. If our hRSs selection pipeline is widely adopted in the field of ART, ROSI technique will also gain broader acceptance. As a result of its extensive application, an increasing number of patients with spermatogenic arrest will benefit from this advancement and have opportunities to father children.

## Materials and methods

### Subjects

Testicular biopsies discarded during routine ART treatment were collected from azoospermia patients treated with mTESE. The investigation had no influence on treatment decisions whatsoever.

### Ethics statement

This study was approved by the Ethics Committee of Peking University Third Hospital (Approval no. M2023849). All of patients recruited in this study were informed with the nature of this study and all of them signed written informed consents.

### Testicular single cell collection and staining

Testicular single cell suspension was collected in accordance with previously published guidelines [21], with some modifications. Seminiferous tubules were minced into a homogeneous suspension by a pair of fine scissors in a 1.5 mL sterile conical tube containing 1 mL G-MOPS™ plus medium (Vitrolife, Vastra Frolunda, Sweden). Next, the suspension was centrifuged at 500 g for 10 min at room temperature. The resulting pellet was then resuspended in 1 mL Accutase solution (Millipore, Massachusetts, USA) with 50 µL Dnase I (5 mg/mL, Thermo Fisher Scientific, Massachusetts, USA) for 15 min at 37 °C. During this period, the suspension was gently pipetted once every 3 min to help it digestion. At the end of this incubation, 1 mL G-MOPS™ plus medium (Vitrolife, Vastra Frolunda, Sweden) containing 5 % human serum albumin was added in the tube to terminate the digestion. The resulting suspension containing spermatogenic cells was filtered through a 70 μm nylon cell strainer (BD Falcon, New Jersey, USA) to remove the tissue debris and then centrifuged at 500 g for 10 min at room temperature. When cells were used to cell isolation by our AI model, the pellet was resuspended in 1 mL of warm G-MOPS™ plus medium and transferred to a 35 × 20 mm dish (CLS430165, CORNING, USA). When cells were used to cell isolation by flow cytometry analysis, the pellet was resuspended in 2 mL of warm G-MOPS™ plus medium with 2 μL Hoechst-33342 (10 mg/ml, Sigma, Missouri, USA), transferred to a 30 mm dish (Thermo Fisher Scientific, Massachusetts, USA) and incubated for 30 min at 37 °C. Before flow cytometric analysis, cell suspension was added PI (2 g/ml, Sigma, Missouri, USA) to exclude dead cells and filtered into a 5 mL polypropylene round-bottom tube with 40 μm nylon cell strainer (BD Falcon, New Jersey, USA).

### Cell sorting by flow cytometric analysis and image capture

Analysis and cell sorting were performed on a dual-laser FACStar Plus flow cytometer (BD FACSAria™ Fusion, BD, New Jersey, USA) equipped with a 355-nm ultraviolet argon laser and a 488-nm argon laser (Coherent, Orsay, France). The FACS instrument settings used to isolate hRSs were adjusted as previous paper [22], [23]. Fluorescence of Hoechst was detected by using a combination of 355-nm long pass and 488-nm short pass filters in front of the first detector. hRSs were isolated according to the brightness of Hoechst blue and the flow-sorted cells were collected in 5 mL polypropylene culture tubes previously coated with about 4 percent of bovine serum albumin (BSA, Sigma, USA). After centrifuging at 500 g for 10 min, the pellet was resuspended in G-MOPS™ plus medium and transferred to a 35 × 20 mm dish (CLS430165, CORNING, USA) for future cell pictures. Under 63x objective magnification and 630x total magnification, cells were observed by invert phase-contrast microscope (Zeiss Observer Z1 Spinning Disk confocal) with detector of Andor EMCCD and immersion medium of IMMERSION OIL (Oil, OLYMPUS, JAPAN) and photographed with numerical aperture of 1.40NA and pixel size of 0.212 µm × 0.212 µm by optical light microscope mounted camera. Meanwhile, diameters of cell and nuclear were examined to get morphological parameters of hRSs.

### Identification of hRSs by immunofluorescence

After sorting by flow cytometry analysis and by our AI model, further identification of hRSs was determined by PNA and/or PRM1 staining. Sorted cell suspension by flow cytometric analysis was centrifuged at 500 g for 10 min at room temperature, and the pellet was resuspended in 1 mL 4 % PFA at 4℃ for overnight. After a fixed cell suspension droplet (5 µl) was deposited on the glass slide coated with polylysine, the area including cells was circled with a water blocking pen to air dried. For cells isolated by our AI model, a glass slide coated with polylysine containing a droplet of 20 µL 4 % PFA was placed on the microscope stage, and 10 isolated cells were aspirated one by one using the micromanipulator from the dish containing testicular single-cell suspension and delivered into the PFA droplet at 4 ℃ for overnight followed by air dry the next day. Immunofluorescence was performed as described [24]. After three washes in phosphate-buffered saline with 0.3 % Triton (PBST), cells were blocked in 5 % BSA at room temperature for 1 h followed by incubation with primary antibodies against PRM1 (1:200, Abcam, USA) and Alexa Fluor 647 conjugated peanut agglutinin (1:400, Sigma, USA) at 4 °C overnight. After three washes in PBST, the sections were incubated with secondary antibody (1:1000, A-11008, Thermo Fisher Scientific, USA) and DAPI (1:1000, ab285390, Abcam, USA) at room temperature for 1 h. After three washes in PBST, the sections were mounted by ProLong™ Glass (P36980, Thermo Fisher, USA). Under 63x objective magnification and 630x total magnification, images with numerical aperture of 1.40NA and pixel size of 0.212 µm × 0.212 µm were captured with a ZEISS LSM880 inverted confocal microscope with detector of Andor EMCCD and immersion medium of IMMERSION OIL (Oil, OLYMPUS, JAPAN). Above immunofluorescence experiments of isolating hRSs by flow cytometric analysis and our AI model were repeated three time, separately.

### Image preprocessing

From the cells collected through the hRSs selection pipeline, images of hRSs obtained via flow cytometric analysis were utilized as the ground truth labels. Referring to these labels, we annotated the images using the widely adopted COCO format, employing an open-source data labeling platform “Label Studio”. In the process of annotating bounding boxes and masks, we implemented an innovative semi-automatic annotation scheme based on segment anything model (SAM). This approach not only ensured high-quality annotations, but also significantly reduced the burden of repetitive labor over time. The entire dataset comprised 3457 images including 8003 hRSs. Following randomization, hRSs samples were divided into a training subset with 6402 hRSs, a validation subset with 800 hRSs and a test subset with 801 hRSs.

To enhance the effectiveness of hRS detection and isolation, all hRS images were z-score normalized using the mean and variance from the training datasets and resized to 1024 × 1024 pixels via bilinear interpolation. These followed some common data preprocessing operations [25], described as follows. These operations were subsequently integrated into our proposed AI model.

1. **Alpha Channel Omitting.** Since transparency was not desired in our work, we omitted the alpha channel.
2. **Image Padding.** Each image was padded at the boarders to maintain a square aspect ratio. Specifically, given an image with shape (w,h), padding was applied along the shorter dimension to equalize it with the longer dimension. Thus, the padded dimensions were changed to (max(w,h),max(w,h)).
3. **Image Resizing.** Each image was then resized to the desired dimensions using bilinear interpolation. The padding applied earlier helped to prevent distortion during the resizing process.
4. **Channel Duplication.** The images were in grey-scale with a single-color channel, which was not compatible to the most pre-trained weights for convolution neural networks (CNNs). Therefore, we duplicated the number of color channels from 1 to 3 to meet this requirement.
5. Color Normalization. Normalizing each color channel is essential to ensure that their distributions remain consistent, thereby facilitating optimal input for the pre-trained weights. To align with the feature-extracted backbone weights pre-trained on ImageNet [26], we applied z-score normalization using a mean of (0.485, 0.456, 0.406) and a standard deviation of (0.299, 0.224, 0.225).

During the training stage, a pipeline of augmentations was employed to further manipulate hRS images in the training subset. It anticipated variations in cell brightness, smoothness, diameter and nuclear perimeter. This data transformed pipeline aimed to ensure that the model obtained after training was robust against these conditions in blind sets. Notably, our training pipeline consisted of several data augmentation methods. Here, we listed the common approaches excluding our proposed strategies:

(1) **Random Vertical Flip.** Each image was vertical flipped with a possibility of 0.5.
(2) **Random Horizontal Flip.** Each image was horizontal flipped with a possibility of 0.5.
(3) **Gaussian Noise.** Each image was noised by a Gaussian noise kernel with a scale of 0.08.
(4) **Gaussian Blur.** Each image was blurred by a Gaussian blur kernel with a sigma of 1.0.

Data augmentation plays a vital role in improving the accuracy and reliability of our artificial intelligence system for identifying hRSs. By applying various image transformations such as flipping, adding slight noise, and adjusting image clarity, we can create additional training examples that mimic real-world variations in cell appearance. This approach was similar to training medical students with diverse case studies – the more varied examples they encounter, the better they become at recognizing conditions in actual clinical practice. These transformations helped our system learn to recognize hRSs even when they appeared under different microscope settings, lighting conditions, or natural biological variations. For instance, cells might appear slightly blurrier in some samples or brighter in others, just as how the same cell type might look different under various microscope settings. This enhanced training made our system more robust and reliable in real-world clinical applications, reducing the chances of misidentification regardless of minor variations in sample preparation or imaging conditions.

## Model selection

### Cascade Mask R-CNN

Mask R-CNN is a widely used deep learning model designed for object detection and instance segmentation tasks. It works by not only identifying objects within an image but also precisely outlining their boundaries (segmentation). The model operates in two main stages: first, it uses a region proposal network (RPN) to locate regions of interest (ROIs) in the image, and then it classifies these regions and generates pixel-level masks for each detected object. In the context of medical imaging, Mask R-CNN can effectively detect and segment structures like cells or tissues, providing clear boundaries for each instance. Its ability to produce accurate and detailed results has made it a popular choice in various biomedical applications.

Cascade Mask R-CNN builds upon the framework of Mask R-CNN, introducing a multi-stage refinement process to improve detection and segmentation performance. It enhances the capacity to model the data distribution of the customized dataset [15], thereby gaining recognition for applications that require higher performance [17], [27]. Mathematically, it takes input images $I\in R^{h\times w\times 3}$. ResNet-101 [28] pre-trained on ImageNet [26] is adopted as the backbone for refining features extracted from different model depths. Multi-scale features are collected and formed into a set of vectors $S=\left\{v:v\in R^{u\times w\times c}\right\}$, where u,w,c vary across different scales. These vectors are then fed into the region proposal network (RPN) to generate pre-selected bounding box areas, termed “proposal”. The RoI heads process these proposals to produce refined results after RoI pooling. As indicated by its name “***cascade***”, each RoI head revises the bounding boxes and segmentation masks from the preceding stages, noting that its weight at each stage is not shared. The output of the entire model consists of three elements: (1) the bounding box of RoI, denoted as $B=\left\{b:b\in R^{6}\right\}$, where the first four values represent its position, and the last two represent its height and width. (2) the segmentation mask, denoted as $M\in {\left[0,1\right]}^{h\times w\times 3}$. A hyperparameter threshold k, along with the thresholding function $t\left(x\right):{\left[0,1\right]}^{h\times w\times 1}\to {\left\{0,1\right\}}^{h\times w\times 1}$, was set to generate the final mask $\hat{M}\in {\left\{0,1\right\}}^{h\times w\times 3}$. (3) the confidence score $s\in \left[0,1\right]$, indicated the confidence of prediction for an object. In summary, multiple stages were cascaded in this model, with each stage refining the results of the previous one. This stepwise approach enhanced the model’s ability to handle challenging cases, such as overlapping objects, small structures, or indistinct boundaries, which were common in medical images like those of hRSs. Each stage in the cascade progressively improved the quality of both object detection and mask segmentation, resulting in higher accuracy and more precise boundaries. This made Cascade Mask R-CNN particularly suitable for tasks requiring detailed and reliable segmentation, such as identifying hRSs within complex microscopy images.

## Training pipeline

### Confusion duplicate & regional drop

We designed a set of innovative data augmentation techniques to enhance the resilience of our AI model. This section was organized into two parts: Motivations and Details. In the Motivations section, we aimed to reinforce the rationale behind our approaches by contrasting them with existing methods. The Details section provided a comprehensive explanation of our design and implementation.

### Motivations

Data augmentation [29], [30], [31], [32] is a commonly used strategy in deep learning to improve a model's ability to generalize to new, unseen data. It involves artificially expanding the training dataset by applying various transformations to the input images, such as rotations, flips, or changes in brightness. However, most existing augmentation approaches treat each image as a whole, modifying the entire image uniformly. While this works well for some tasks, it is often ineffective for dense prediction tasks, such as detecting and segmenting objects like hRSs. This is because only a small portion of the image, typically the target regions (such as hRSs), contains useful information, and focusing on the entire image may dilute the model's attention to these critical areas.

To address this limitation, some prior methods have aimed to augment images by focusing on specific assumptions [33], [34] about the data. However, these approaches often lack consistency and stability compared to techniques that rely on supervised training. For tasks requiring precise detection and segmentation, we believe it is essential to use robust supervision that enhances the representation of critical regions in the images. For example, Diao et al. proposed a method [19] that duplicates the positive regions in an image—areas that contain the objects of interest, such as hRSs—to increase their representation during training. This method proved effective in helping the model focus more on relevant areas.

Building on this idea, we proposed that not only the ground truth (accurately labeled regions) but also FP and FN samples should be included during augmentation. FPs were regions incorrectly identified as hRSs, while FNs were actual hRSs that the model failed to detect. By incorporating these FP and FN regions into the training process, the model can learn to better distinguish hRSs from other regions and reduce errors. To achieve this, we developed a pipeline that systematically identified FP and FN samples, duplicated them, and pasted them into input images during training. This tailored augmentation approach ensured that the model got exposed to a more diverse range of examples, ultimately improving its ability to accurately detect and segment hRSs in challenging scenarios.

### Details

**Fig. 1:**
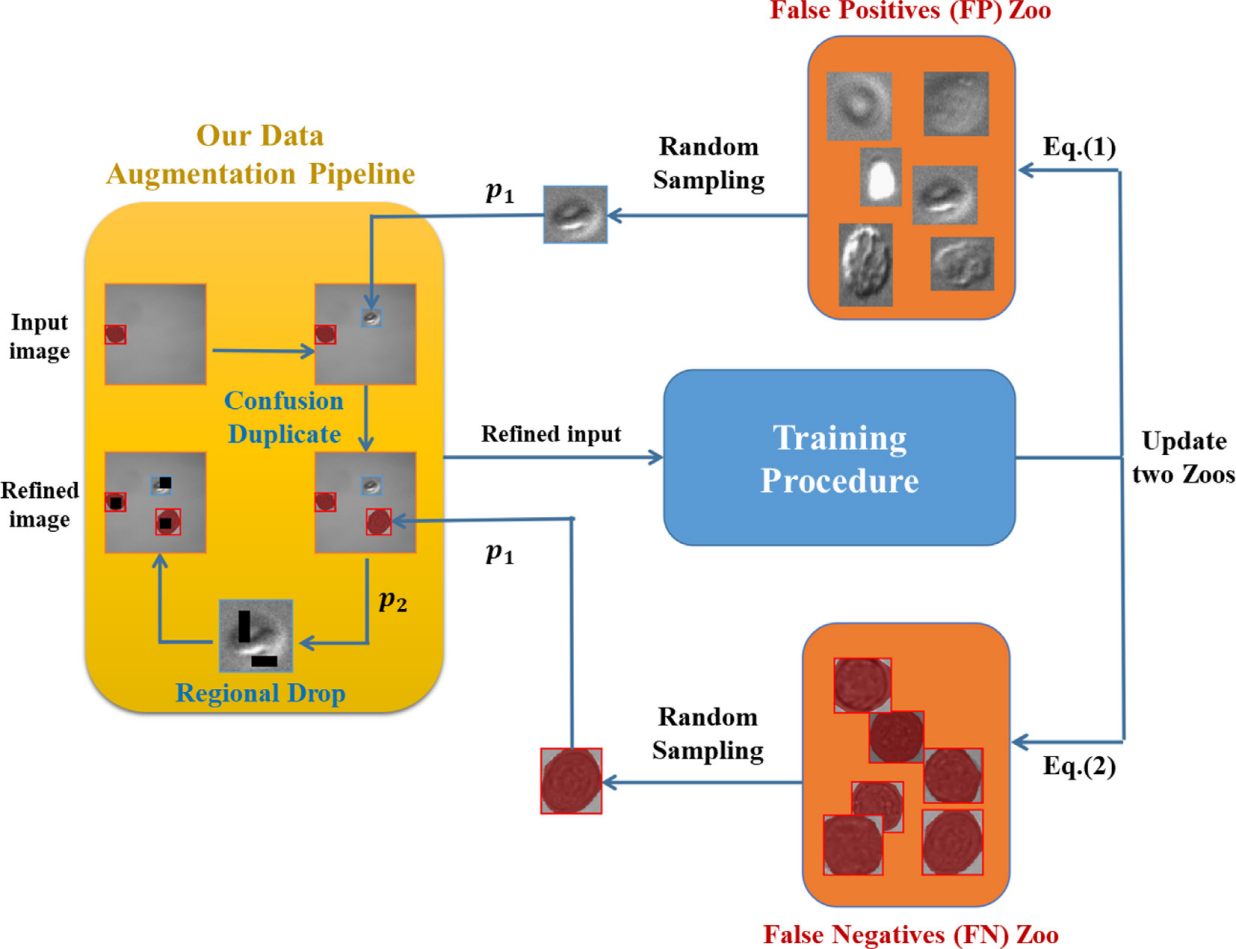
The overview of the training process incorporating our proposed data augmentation pipeline. Additional objects to be added into the input image were randomly sampled with a possibility $p_{1}$ from both FP Zoo and FN Zoo. Subsequently, the image had a possibility $p_{2}$ of undergoing Regional Drop. The output from each training iteration was used to update above two sets. Better viewed in color.

To this end, we maintained a collection referred to as the False Positive Zoo (FP Zoo) to restore the FP regions during training, as well as a set termed False Negative Zoo (FN Zoo) (Fig. 1). This operation was named as “Confusion Duplicate”. Next, we discussed the construction process in detail.

A false positive (FP) occured when the model incorrectly identified something as a hRS when it was not. For example, in microscopy images, the model might mistakenly detect debris, other cell types, or background artifacts as hRSs. These errors can lead to overestimation of the number of hRSs present, potentially causing confusion or inaccurate conclusions during analysis. In simpler terms, a false positive was like a false alarm where the model saw something that was not actually relevant. Minimizing false positives was crucial to ensure that the detection results were meaningful and reliable for clinical or research applications. Thus, at each training step, we first identified the intersection of the ground truth and our predictions that felled below the confidence threshold. We then assessed whether the FP Zoo exceeded its maximum allowable size to determine how to update it, as described mathematically below:

(1) $${\mathrm{FP}}_{\mathrm{ct}}^{n}=\left\{\begin{array}{c} {\mathrm{FP}}_{\mathrm{ct}}^{n-1}\cup \left(G\cap \left\{P:c\left(P\right)<ct\right\}\right)/\left\{z\right\},card\left({\mathrm{FP}}_{\mathrm{ct}}^{n-1}\right)=T\\ {\mathrm{FP}}_{\mathrm{ct}}^{n-1}\cup \left(G\cap \left\{P:c\left(P\right)<ct\right\}\right),card\left({\mathrm{FP}}_{\mathrm{ct}}^{n-1}\right)<T \end{array}\right)$$

where G represented the ground truth of the given image, P denoted the output RoI, $c\left(∙\right)$ was the function utilized to determine an object’s confidence score, and ct was the threshold for $c\left(∙\right)$. z was the first element entered in $Z_{\mathrm{FP}}^{t-1}$, T was the maximum capacity of the FP Zoo, $card\left(∙\right)$ indicated the sample amount in the Zoo, and n denoted the training step number.

Meanwhile, a false negative (FN) happened when the model failed to detect a hRS that was actually present in the image. For instance, the model might overlook small or faintly visible hRSs due to their size, shape, or poor contrast in the image. This type of error can lead to an underestimation of the hRS count, potentially missing important biological or diagnostic information. In essence, a false negative was like a missed detection, where the model “ignores” something it should have identified. Reducing false negatives was vital to ensure that no important hRSs were overlooked during the analysis. Thus, for updating the FN Zoo, we considered those predictions that exceeded the specified confidence threshold but did not overlap with any ground truth positive region by more than the threshold γ. The FN Zoo was described mathematically as follows.

(2) $${\mathrm{FN}}_{\mathrm{ct}}^{n}=\left\{\begin{array}{c} {\mathrm{FN}}_{\mathrm{ct}}^{n-1}\cup \left(\left(I\backslash G\right)\cap \left\{P:c\left(P\right)>ct\right\}\right)\backslash \left\{z\right\},card\left({\mathrm{FN}}_{\mathrm{ct}}^{n-1}\right)=T\\ {\mathrm{FN}}_{\mathrm{ct}}^{n-1}\cup \left(\left(I\backslash G\right)\cap \left\{P:c\left(P\right)>ct\right\}\right),card\left({\mathrm{FN}}_{\mathrm{ct}}^{n-1}\right)<T \end{array}\right)$$

where I represented the whole image. By updating these two sets, we randomly selected objects from ${\mathrm{FP}}_{\mathrm{ct}}^{n}$ and ${\mathrm{FN}}_{\mathrm{ct}}^{n}$ with a possibility $p_{1}$ to incorporate into the current image I.

**Fig. 2:**
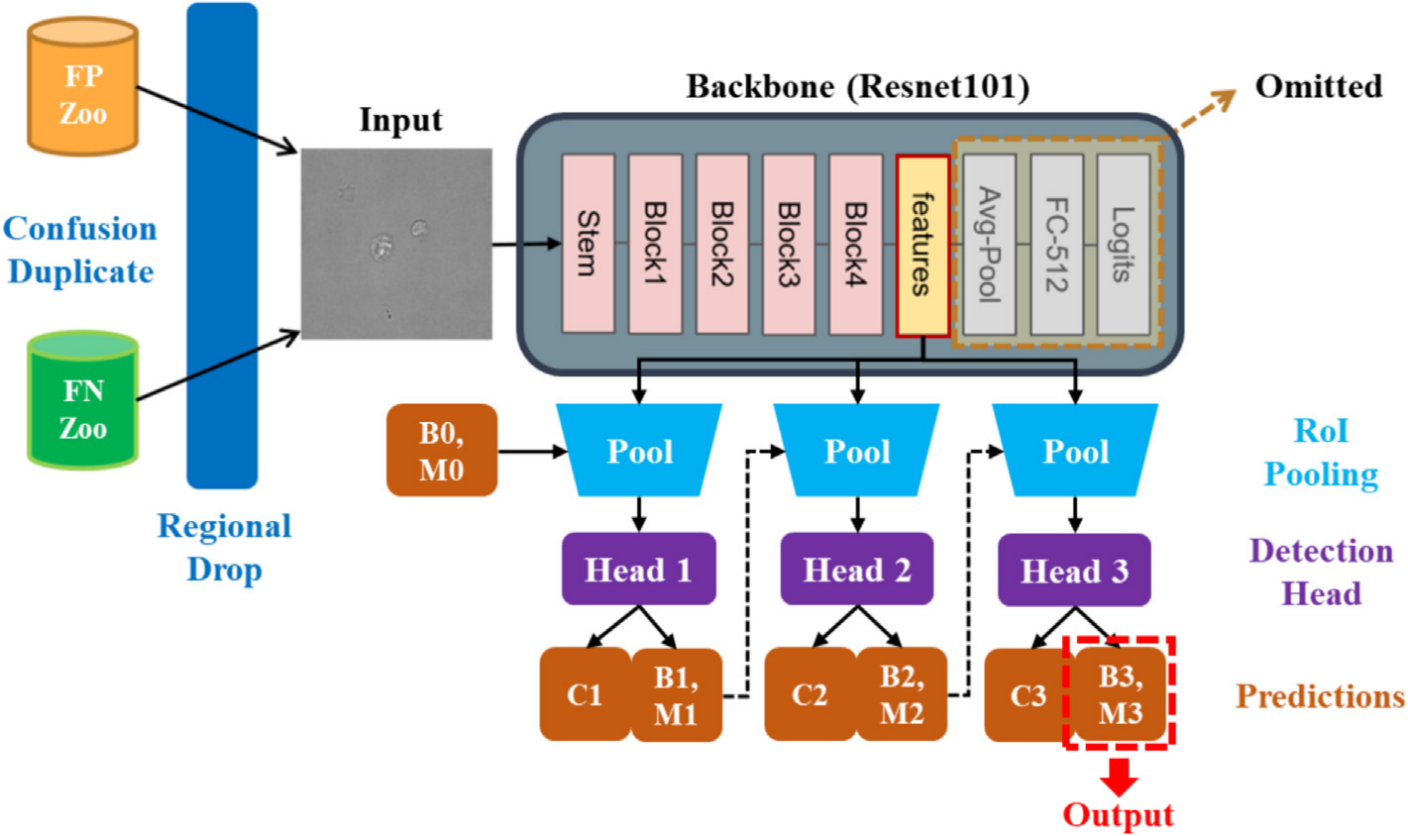
Our model architecture. Given an input image, it first underwent our designed data augmentation techniques, specifically Confusion Duplicate & Regional Drop, to obtain a refined image. This pre-processed image is then fed into the feature-extracted backbone (ResNet-101 in our case) with the classification head omitted. The Cascade Mask R-CNN decoder was then attached, enabling the model to receive features at different scales. The detection module subsequently generated predictions for classes, bounding boxes and masks. The predictions of the final stage were treated as the output of the entire model.

To further alleviate the risk of overfitting and enhance generalization, we randomly set k×k blocks to be zero in the ground truth, FP and FN regions, with a possibility $p_{2}$. This operation termed as “Regional Drop”, inspired by the previous report [35], suggested that regional dropping was more effective than pixel-level dropping in the context of computer vision tasks. The complete proposed pipeline of our data augmentation strategies was illustrated in Fig. 2.

While the training loss function l was minimized, the expectation L was concurrently minimized as follows:

(3) $$L=E_{I_{i},y_{i}\;h\left(I,y\right),I_{FP},y_{FP}\;h\left(Z_{\mathrm{FP}}^{n}\right),I_{FN},y_{FN}\;h\left(Z_{\mathrm{FN}}^{n}\right)}l\left({q(I}_{i},I_{FP},I_{FN}\right),q\left(y_{i},y_{FP},y_{FN}\right))$$

where h(∙) represented a uniform distribution over the entire datasets, q(∙) denoted the proposed data augmentation pipeline, and y was the ground truth of image I. From Eq. (3), it was evident that FP and FN played a significant role in the optimization target, which was the primary objective behind the design of our data augmentation approach.

### Training implementation

We employed Cascade Mask R-CNN [15] with the ResNet-101 [28] backbone, and loaded pre-trained weights on ImageNet [26] to facilitate faster convergence. According to the cascade architecture, we implemented three cascade stages for the RoI head. The AdamW [36] optimizer was chosen with a weight decay of 0.05, eps of 1e-8, and betas of (0.9, 0.999). The learning rate was initialized from 0 to 1e-3 in warmup style [37] during the first 500 iterations (at batch level), then decaying in a cosine curve [38]. Our proposed Confusion Duplicate & Regional Drop were applied through the whole training process. Both the FP Zoo and FN Zoo were constrained to a maximum size of 10. The probabilities $p_{1}$ and $p_{2}$ were both set to 0.5. Based on our pilot study, our AI model converged in approximately 30 epochs, resulting in the maximum training epoch being set to 30.

### Cell sorting by AI model and subsequently identification by immunofluorescence

Human testicular cell suspension containing various types of spermatogenic cells was made a droplet media which was covered mineral oil (Ovoil, Vitrolife, Göteborg, Sweden) in a 60-mm culture dish (FALCON353652, Corning, New York, USA) under an inverted phase-contrast microscope. When assumed hRSs were noninvasively identified by our AI model at × 630 magnification, they were aspirated carefully and moved to another droplet media by micro pipette. After assumed hRSs were aspirated carefully and moved to a 20-μl microdroplet media of 4 %PFA on a glass slide by micro pipette, immunofluorescence staining was performed as described in the above section. Three repeated experiments as described above have been done.

## Results

### Isolation and identification of hRSs by flow cytometric analysis

**Fig. 3:**
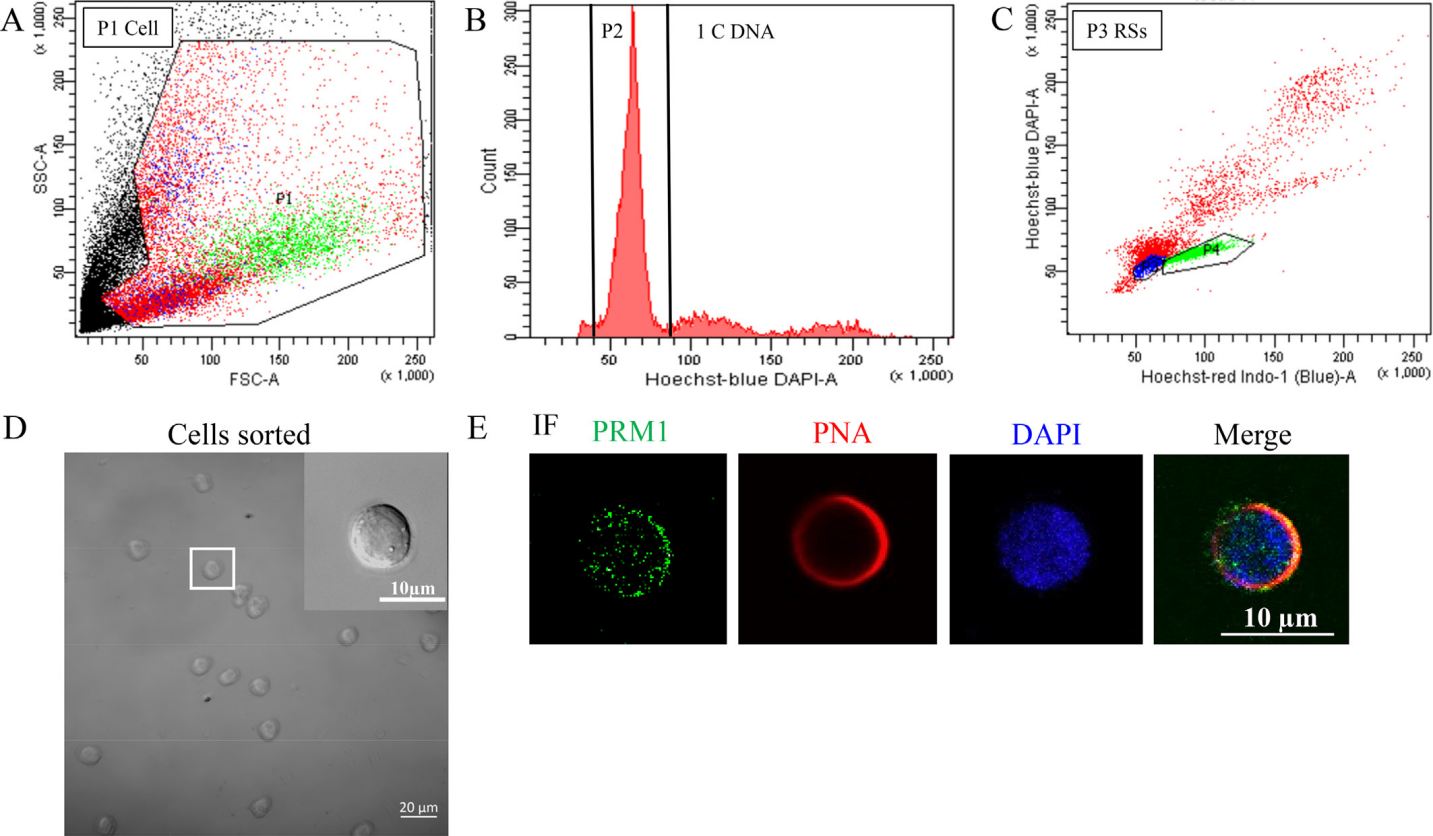
Flow cytometric analysis of human testicular cell populations based on Hoechst and PI fluorescence and light scattering parameters and identification of sorted cells. A) Scatter plot of FSC and SSC. Cells and debris were separated based on scattering parameters including cell size (FSC) and cell granularity (SSC). P1 region included all cell population and other region containing debris was excluded. B) Analysis diagram of DNA content based on Hoechst fluorescence. P2 region included haploid cells with 1C DNA content. C) Scatter plot of fluorescence intensity. hRSs population was identifiable in the P3 region (blue region). D) Morphology of sorted cell. These cells showed typical morphological characteristics of hRSs. Scale bars, 10 µm. E) Immunofluorescence of PNA and PRM1 in sorted cells by flow cytometric analysis. Scale bars, 10 µm. hRSs, human round spermatids; FSC, forward scatter; SSC, side scatter.

Debris were excluded by excluding low FSC intensity signals (Fig. 3A). Haploid hRSs from P3 region were sorted and collected based on the Hoechst fluorescence profile (Fig. 3B&C) which was consistent with previous papers [23], [39]. For cells isolated by flow cytometric analysis, the average cell diameter, nuclear diameter and ratio of nucleus to cytoplasm were about 11 μm, 7 μm and 0.3 separately which were similar with published lectures[23], [40]. Meanwhile these sorted cells had typical characteristics of hRSs including round cells, spherical nucleus, one nucleolus in one cell and acrosomal caps in most cells (Fig. 3D). Expression of PNA-647 and/or PRM1 (RSs markers) confirmed that these sorted cells were hRSs (Fig. 3E).

## Algorithm evaluation

### Evaluation metrics

We selected mAP as the evaluation metric due to its ability to balance both precision and recall. Besides, we clarified the mAP calculation to ensure that our work was both clear and convincing. The model generated predictions of both the corresponding bounding boxes and masks. Each prediction can be represented as a set s={p,b,m}. Here, p∈R was the confidence score ranging from 0 to 1, $b\in R^{4}$ represented coordinates of the left-top and right-bottom corners of a bounding box, $m\in {\left\{0,1\right\}}^{h\times w}$ denoted the mask used to indicate labels of each image pixel, determining whether it corresponded to a hRS. To compute mAP, we first sorted the set s in descending order based on the values of p. The mAP value can be expressed as follows:

(4) $$\mathrm{mAP}=\sum_{i}^{N}\sum_{c}^{C}\frac{\mathrm{Pr}\left(s_{\mathrm{ic}}\right)*I\left(s_{\mathrm{ic}}\right)}{\sum_{t}^{C}I\left(s_{\mathrm{it}}\right)}$$

where $\mathrm{Pr}(s_{\mathrm{ic}})$ represented the precision of the first c predictions of the i-th image, $I(s_{\mathrm{ic}})$ was the indicator function which outputted 1 when c-th prediction was correct and 0 otherwise. For a single predicted instance, if the overlapping area was greater than half of union area of the prediction and ground truth, it was considered a match. N was the total amount of evaluated images. This formulation as described above was based on the assumption as follows:

(5) $$\sum_{c}^{C}\frac{\mathrm{Pr}\left(s_{\mathrm{ic}}\right)*I\left(s_{\mathrm{ic}}\right)}{\sum_{t}^{C}I\left(s_{\mathrm{it}}\right)}=0,if\sum_{t}^{C}I\left(s_{\mathrm{it}}\right)>0\;\;$$

### Comparison with other State-of-The-Art methods

To assess the effectiveness of our AI model, we compared our augmentation strategies with several state-of-the-art augmentation techniques. In the field of deep learning for medical image analysis, various data augmentation methods have been developed to improve the performance and generalization ability of models. In this study, we selected Mixup[41], Cutout[42], Dropblock [35], Mosaic [43], and RandomErasing[44] for comparison with our proposed method. The reasons were as follows. Firstly, these methods were widely used and have shown good performance in previous studies. They can effectively increase the diversity of the training data, which was beneficial for the model to learn more robust features. For example, Mixup can combine different images to generate new samples, and Cutout randomly removed parts of the image to force the model to focus on other regions. Mosaic combined four different images into one, which enriched the training data and helped the model learn different features from multiple samples simultaneously. RandomErasing randomly selected a rectangular area in the image and erased its pixels. This forced the model to learn more robust features, making the model less sensitive to the presence or absence of specific regions and potentially improving its accuracy in identifying the targets. Secondly, these methods were relatively simple and easy to understand, which was suitable for our research context where we needed to communicate with non-technical audiences. Comparing our method with these established techniques, we can better evaluate the effectiveness and superiority of our proposed approach in the task of identifying hRSs. This comparison will provide more comprehensive and reliable results, which is of great significance for the development and application of our method in the field of reproductive medicine.

To ensure a fair and thorough evaluation of our model's performance, we conducted extensive testing using multiple approaches and standardized testing conditions. We compared our method with several existing approaches, carefully adjusting each method's settings to achieve its optimal performance. This process was similar to how different medical diagnostic tools might need individual calibration while following the same overall testing protocol. Each method was evaluated through repeated testing cycles, and we selected the best-performing version for final assessment. To validate the reliability of our results, we repeated each test three times under different initial conditions – much like how clinical trials often require multiple rounds to confirm findings. The results, presented in Table 1, showed that our method consistently achieved higher accuracy compared to other approaches. Importantly, our model maintained stable performance across different test runs, demonstrating reliable reproducibility – a crucial factor in clinical applications.

**Table 1:** Comparison with other state-of-the-art augmentation methods.

Model	Augmentation Methods	Bbox mAP (%)	Mask mAP (%)
Cascade Mask R-CNN	None	0.737 ± 0.003	0.743 ± 0.003
	Mixup	0.740 ± 0.004	0.744 ± 0.004
	Cutout	0.748 ± 0.004	0.753 ± 0.004
	Dropblock	0.788 ± 0.007	0.791 ± 0.007
	Mosaic	0.785 ± 0.006	0.790 ± 0.006
	RandomErasing	0.775 ± 0.005	0.777 ± 0.005
	Ours	**0.800**±**0.006**	**0.797**±**0.006**

The results are based on the average mAP (%) of three runs per experiment. The standard errors are reported as well for clarifying the robustness. The best numbers are highlighted in **bold** font.

**Fig. 4:**
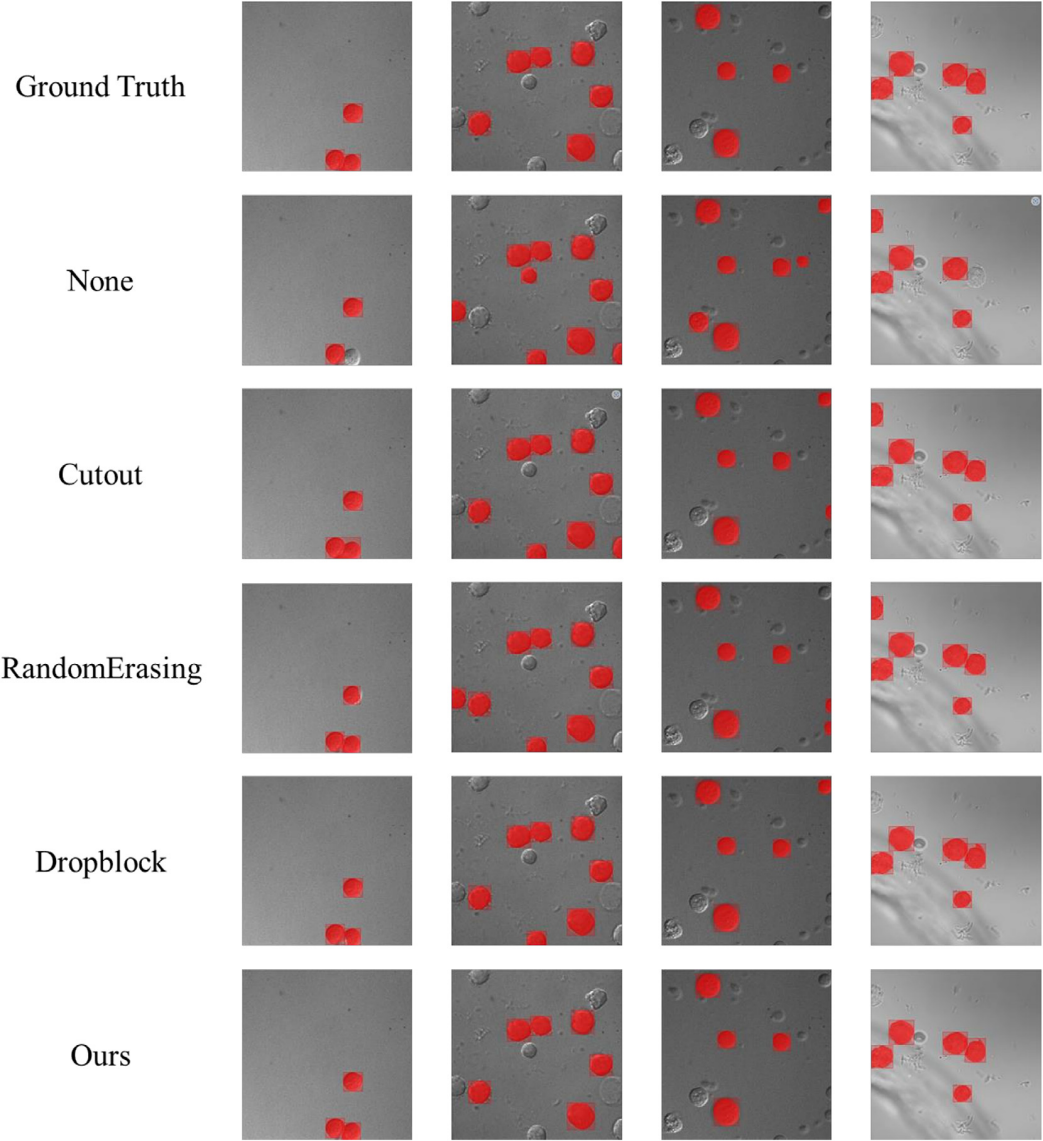
The visualization predictions of our method and other augmentation methods on the test subset. Our method not only has a high accuracy in hRSs recognition, but also has high quality in predicting detection boxes and masks.

The visual comparison of the prediction results between the above methods and our proposed in this article was shown in Fig. 4. Mixup and Cutout did not significantly improve the performance of the vanilla model, likely due to the high sparsity of the data. Dropblock, which randomly blanked out blocks in the intermediate feature maps, exhibited a few boosts in mAP as the information of small hRSs was diffused to each block. Mosaic combined multiple images and RandomErasing randomly erased rectangular areas in the image, both aiming to enhance the model's learning ability. Overall, our method, which took into account the RoI sparsity nature, achieved the best performance. However, it came with a relatively high standard error, similar to the levels of other augmentation methods. While our method showed superiority in identifying hRSs, there was still room for improvement in terms of stability. The comparison results provided valuable insights into the effectiveness of different data augmentation strategies for this specific task.

**Fig. 5:**
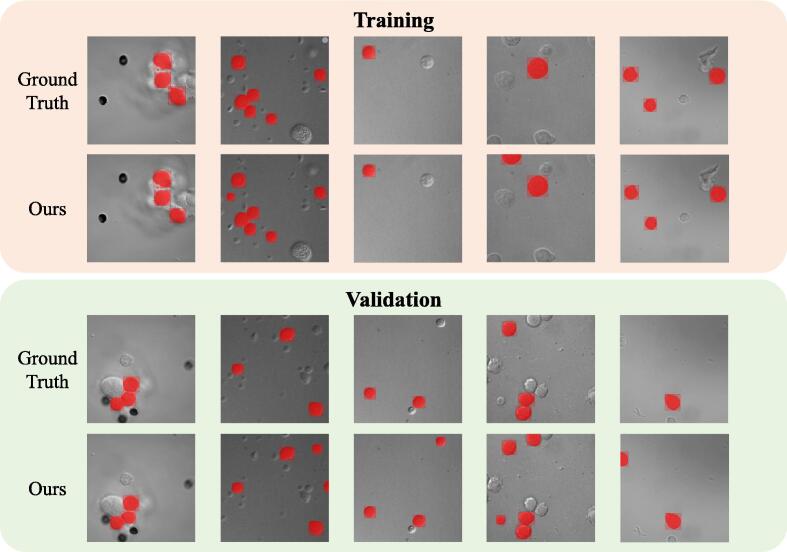
The visualization of the prediction results of our method on the training subset and validation subset. Since the model parameters are trained on the training subset, the quality of the prediction results of the training set is higher than that of the validation subset.

**Fig. 6:**
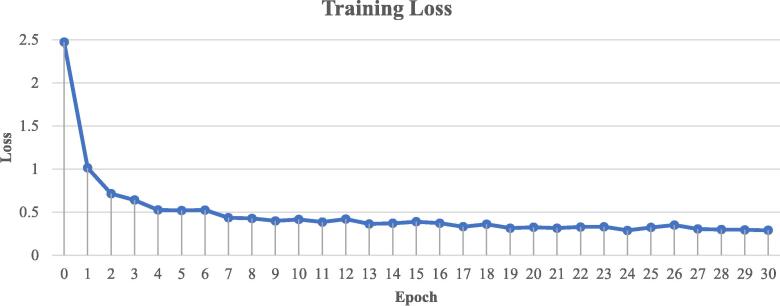
The trend of loss changes during model training on the training subset. At epoch 30, the training loss has basically converged.

As shown in Fig. 5, our AI model demonstrated consistent and reliable performance across the training and validation subsets. It performed particularly well with images it was initially trained on, which was expected as these images were used to develop its recognition capabilities. This was similar to how medical professionals typically perform better at diagnosing conditions they have encountered frequently during their training. When tested on new, previously unseen images, it maintained strong performance, though slightly lower than with familiar images. We monitored the learning progress over time and observed that it showed significant improvement in accuracy during the first 30 learning cycles. After this period, the improvement plateaued, suggesting that additional training would not yield meaningful benefits. This learning pattern was analogous to how medical residents typically showed rapid improvement in their diagnostic skills during initial training before reaching a stable level of competency. Based on these observations in Fig. 6, we determined that 30 learning cycles were optimal for achieving reliable and consistent results in identifying hRSs.

### Ablation study

**Fig. 7:**
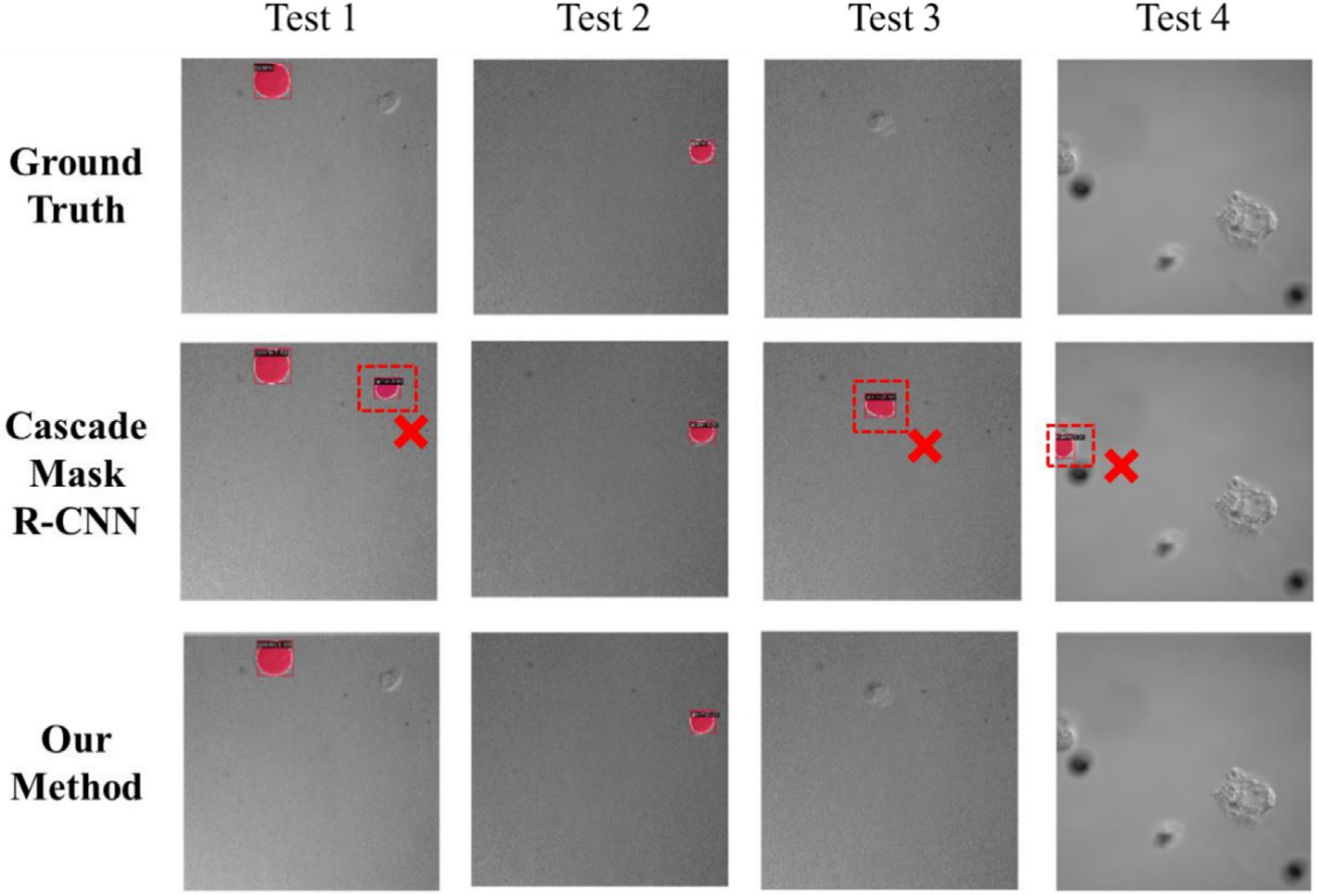
A qualitative visualization of our method and Cascade Mask-RCNN. The red boarders around the marked regions denoted the ground truths and predictions, respectively. We excluded regions with confidence scores below 0.1 from the visualization. Better viewed in color.

To better understand how each component of our novel method contributes to its overall effectiveness, we conducted a systematic evaluation process, similar to how medical researchers might study the individual effects of different components in a combination therapy. We started by testing a basic version of our model, then gradually added new features one by one to measure their individual impact. As shown in Table 2, each added component improved the model's accuracy for identifying hRSs. Our two key innovations, a method to handle confusing cases and a technique to focus on specific regions, were proved particularly effective in enhancing the model's performance. To provide a clear understanding of these improvements, we created visual comparisons (Fig. 7) showing how our enhanced model performed compared to the basic version. This comprehensive improvement resulted in our model achieving an accuracy rate of 80 %, making it a reliable tool for clinical applications. This systematic evaluation approach ensured that each component of our method served a valuable purpose in improving overall accuracy and reliability.

**Table 2:** Ablation study on our proposed method with different module combination.

Description	+FN Zoo	+FP Zoo	+RoI-Drop	Bbox mAP (%)	Mask mAP (%)
Cascade Mask R-CNN				0.737 ± 0.003	0.743 ± 0.003
	√			0.778 ± 0.004	0.778 ± 0.004
	√	√		0.791 ± 0.004	0.795 ± 0.004
	√	√	√	**0.800**±**0.006**	**0.797**±**0.006**

The results are based on the average mAP (%) of three runs per experiment. The standard errors are reported as well for clarifying the robustness. The best numbers are highlighted in **bold** font.

To further validate the versatility and effectiveness of our improved method, we extended our testing to include different artificial intelligence systems commonly used in medical image analysis. This approach was similar to testing a new medical protocol across different hospital settings to ensure its broad applicability. We wanted to determine whether our enhanced image processing techniques would improve the performance of other existing systems, not just our primary system. The results of our proposed data augmentation techniques with other instance segmentation base models instead of Cascade Mask R-CNN, detailed in Table 3, demonstrated that our improvements consistently enhanced the performance of various image analysis systems. This broad success across different platforms was particularly important for clinical applications, as it suggested that our methods can be effectively integrated into various existing medical imaging systems, providing flexibility for different clinical settings and requirements. Just as a successful medical treatment should work effectively across different patient populations, our improvements proved beneficial regardless of the underlying analysis system used. These comprehensive results strengthened our confidence in recommending these techniques for widespread adoption in clinical practice.

**Table 3:** Ablation Study on our proposed method with different base models.

Method	Bbox mAP (%)	Mask mAP (%)
Mask R-CNN[50]	0.777 (+0.065 ↑)	0.776 (+0.063 ↑)
PointRend [51]	0.792 (+0.060 ↑)	0.792 (+0.059 ↑)
SCNet [52]	0.807 (+0.045 ↑)	0.809 (+0.044 ↑)
DetectoRS[53]	**0.812** (+0.037 ↑)	**0.813** (+0.037 ↑)
Our method	0.800 (+0.063 ↑)	0.797 (+0.054 ↑)

The results are based on the average mAP (%) of three runs per experiment. The standard errors are reported as well for clarifying the robustness. The best numbers are highlighted in **bold** font.

### Identification of hRSs by AI model and immunofluorescence

**Fig. 8:**
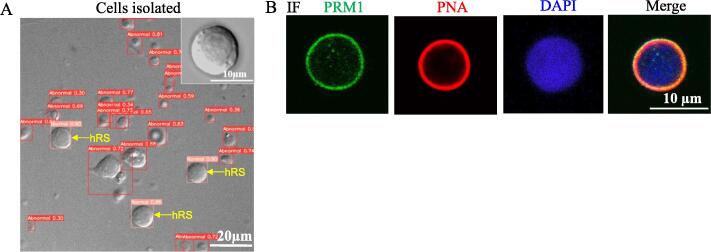
Selection and identification of presumptive hRSs by our AI model. A) Single cell suspension and presumptive hRSs isolated by our AI model (arrowheads). Scale bars, 20 µm. B) Immunofluorescence of PNA and PRM1 in sorted cells by our AI model. Scale bars, 10 µm.

Testicular cell suspension was observed under an inverted phase-contrast microscope. Cells labeled as “Normal” were identified as hRSs by our AI model (Fig. 8A). The expression of PNA and/or PRM1 in all 30 presumed hRSs identified by our AI model through immunofluorescence staining confirmed that these cells were indeed hRSs (Fig. 8B). Comparison of accuracy results between vanilla Cascade Mask-RCNN and our AI models for identifying hRSs demonstrated that our AI model had higher accurate than vanilla Cascade Mask-RCNN model. There were two out of 12 cells sorted by vanilla Cascade Mask-RCNN model which were negative in PRM1 and PNA. On the contrary, all of cells screened by our AI model expressed PRM1 and PNA (Supplemental figure 1).

## Discussion

hRSs differentiated from second meiosis of the spermatogonium have the same number of chromosomes (haploid DNA) as those of mature sperm, so these cells can be used to injected into oocytes followed with proper oocyte activation technique to get fertilized oocytes and embryos. First baby was born by ROSI technique almost 30 years ago [5], but there were just several reports about the clinical application of ROSI [4], [6], [7], [8], [9]. To data, just one reproductive center could extensively apply this technique in clinical treatment, and 90 babies were born from ROSI. Poor reproducibility, main drawback of currently method for identifying hRSs which depends on the experiences of embryologists based on their morphology and physical characteristics, is the main reason to restrict the widespread application of ROSI technique in the clinic. Thus, most of these NOA patients demonstrating above clinical phenotype can’t get effective therapeutic procedures.

Some previous studies showed that RSs were precisely screened by flow cytometric analysis based on the Hoechst fluorescence profile reflecting DNA ploidy [23], so animal off-springs born from ROSI using RSs isolated by flow cytometric analysis including mice and boars have been reported [45], [46]. However, Hoechst and ultraviolet light are harmful to DNA and could induce slight toxicity, moderate mutation and significant cell cycle perturbation of human spermatogenic cells [47]. Therefore, this invasive method requiring DNA staining can’t be applicable in clinical practice for isolating hRSs.

AI has been widely applied in the field of clinical application and has gradually demonstrated its unique advantages and precision. In this study, we developed a deep learning AI model combined with innovative data augmentation strategies for accurately and noninvasively identifying hRSs. A large number of hRSs images isolated by flow cytometry analysis were used to construct the model, and this model subsequently was utilized to noninvasively identify hRSs in testicular single cell suspension. The final double-blind experiment proved that our AI model could 100 percent noninvasively identify hRSs. Although tremendous focus has been put on the design of the model structure in AI-based medical imaging field [48], [49], heuristic design during the data augmentation process is vital based on our work. Moreover, such heuristic design made these methods intuitive and convincing.

Invert phase-contrast microscope was used to observe assumed hRSs identified by our AI model and these cells showed typical characteristics of hRSs including size (～10 μm) and morphology. The morphological characteristic of assumed hRSs selected by our AI model was in line with previous studies [4], [40]. Furthermore, immunofluorescence results of assumed hRSs demonstrated that all of they were hRSs due to their expression of PNA and/or PRM1 (RSs markers). Therefore, above double-blind experiment made further efforts to prove that our AI model was precise, effective and specific. The major obstacle hindering the widely clinical application of ROSI treatments is the unambiguous identification of hRSs, and our AI model successfully resolved the above obstacle. Furthermore, long-term validation studies related to clinical outcomes such as fertilization and blastocyst formation rates would be needed to go a step to validate the specificity and precision of our model in the real-world applications of ROSI. And this is something we are actively pursuing in our subsequent research. We plan to collaborate with clinical experts to apply our AI model to clinical research and monitor clinical outcomes over extended periods.

Comparing traditional method for isolating hRSs, this model has several obviously noteworthy advantages needed to be pointed out. Firstly, there is no doubt that this model for identifying hRSs could be used to precise screen hRSs. Expression of PRM1 and/or PNA in assumed hRSs isolated by our model have confirmed the precision and specificity of this model. Secondly, this method is non-invasive. Testicular cells were neither stained nor any other extra processed. Thirdly, this method is simple and repeatable. Anyone can use it for isolating hRSs so long as he/she has this model. Finally, this method is very efficient. Embryologists don’t need spend so long time to recognize hRSs because identification of hRSs don’t depend on work experiences. The model will figure out which one is indeed a hRS in a few seconds. If it could be widely used in clinical field for selecting hRSs to treat NOA patients with spermatogenic arrest at the hRS stage, ROSI would be used by more and more embryologists and become a more practical treatment for such NOA patients.

## Conclusion

In conclusion, we developed a novel deep learning AI model which could be used to noninvasively and accurately isolate hRSs in testicular single cell suspension. Our model is very valuable and practical for promoting widely clinical application and rapidly development of ROSI technique throughout the world.

## Source of funding

This study was supported by the Natural Science Foundation of Beijing Municipality (7242164) and Innovation Transformation Fund received from the Peking University Third Hospital (BYSYZHKC2023103). The content is solely the responsibility of the authors. The authors declare no competing interests.

## Ethical approval and consent to participate

This study was approved by the Ethics Committee of 10.13039/501100009399Peking University Third Hospital (Approval no. M2023849).

## Declaration of competing interest

The authors declare that they have no known competing financial interests or personal relationships that could have appeared to influence the work reported in this paper.
