# Supplementary material for: Development and validation of a deep learning model based on cascade mask regional convolutional neural network to noninvasively and accurately identify human round spermatids
Source: J Adv Res. 2025 Apr 2;79:737–49. doi: 10.1016/j.jare.2025.03.059 (PMC12766188; doi:10.1016/j.jare.2025.03.059)
Supplement: Supplementary Data 1 [file mmc1.pdf]

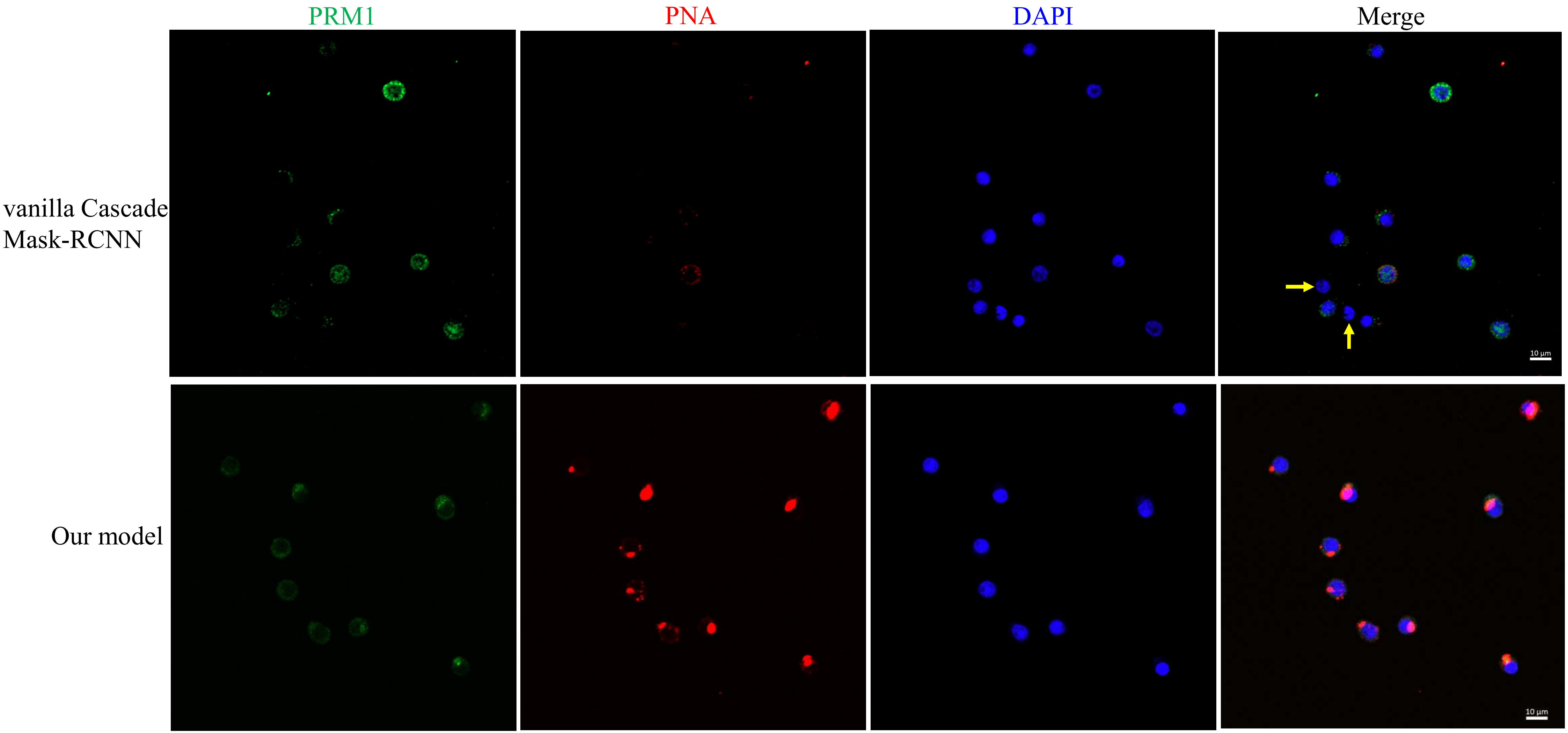


Supplemental figure 1. Immunofluorescence of PNA and PRM1 in sorted cells by vanilla Cascade Mask-RCNN versus our AI models. Yellow arrowheads showed the non-hRSs isolated by vanilla Cascade Mask-RCNN model. Scale bars, 10μm.
